# Supplementary figures and images for: Drosophila eIF3f1 mediates host immune defense by targeting dTak1
Source: EMBO Rep. 2024 Jan 26;25(3):26. doi: 10.1038/s44319-024-00067-z (PMC10933477; doi:10.1038/s44319-024-00067-z)

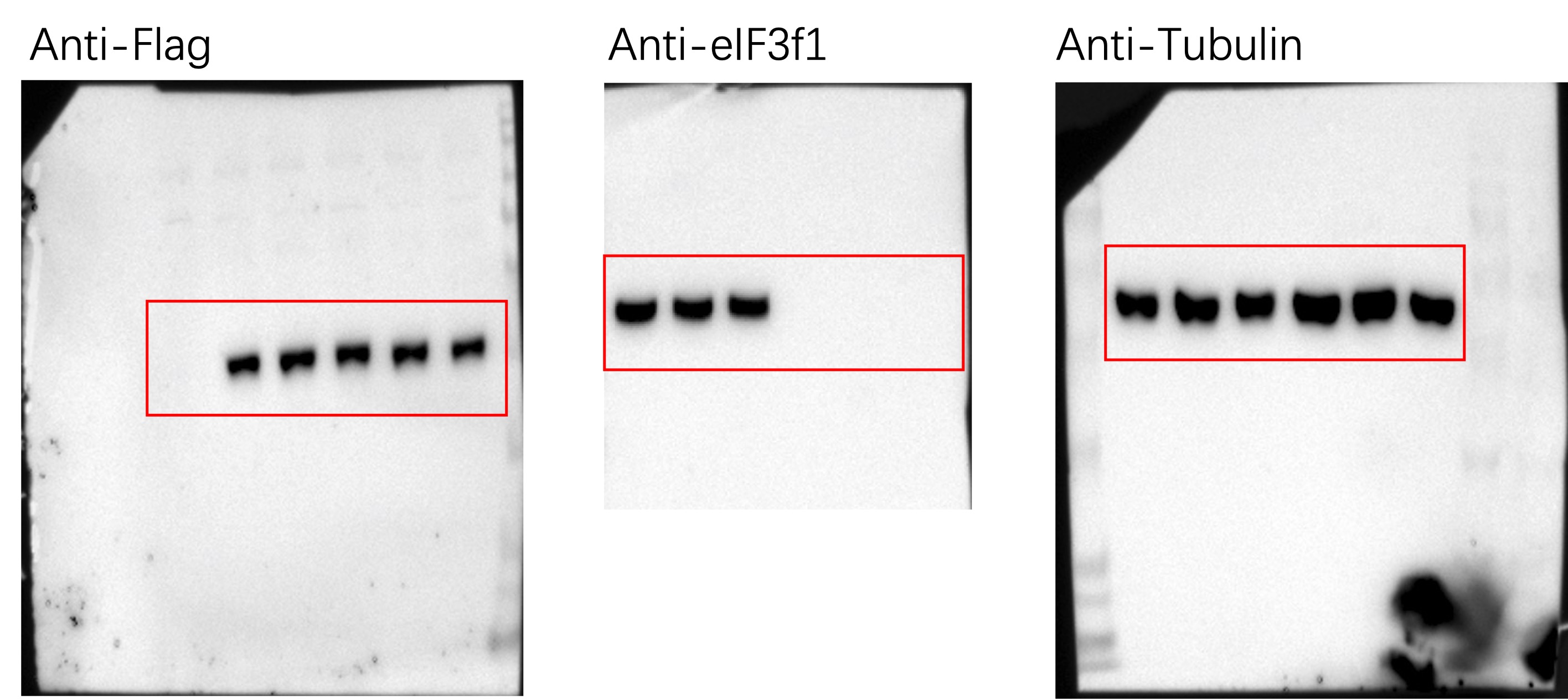

Supplement: Supplementary file 1 — Source Data Fig. 1 [file 44319_2024_67_MOESM1_ESM.zip › Figure 1/1A/Western blot.jpg]

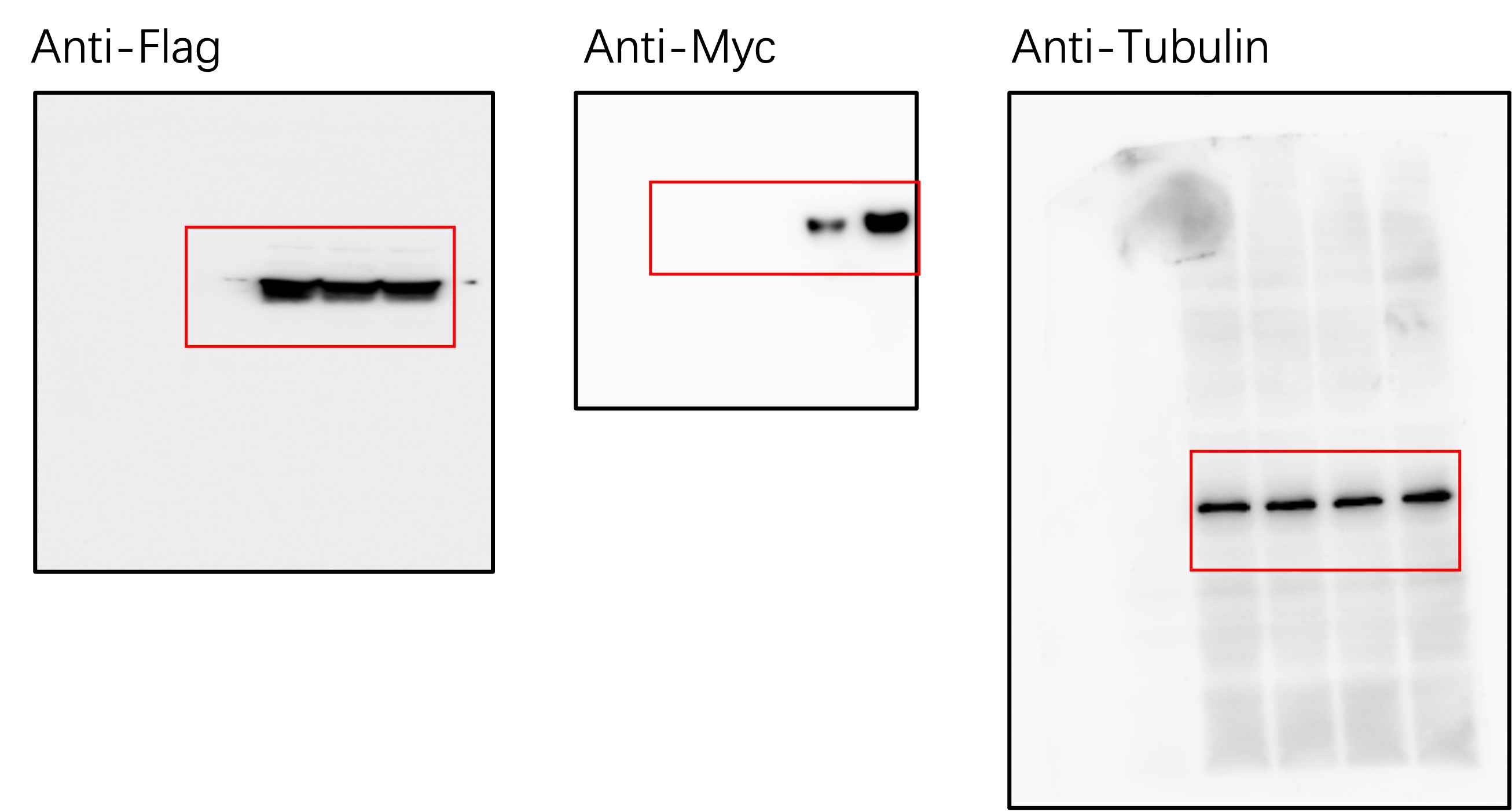

Supplement: Supplementary file 1 — Source Data Fig. 1 [file 44319_2024_67_MOESM1_ESM.zip › Figure 1/1E/Western blot.jpg]

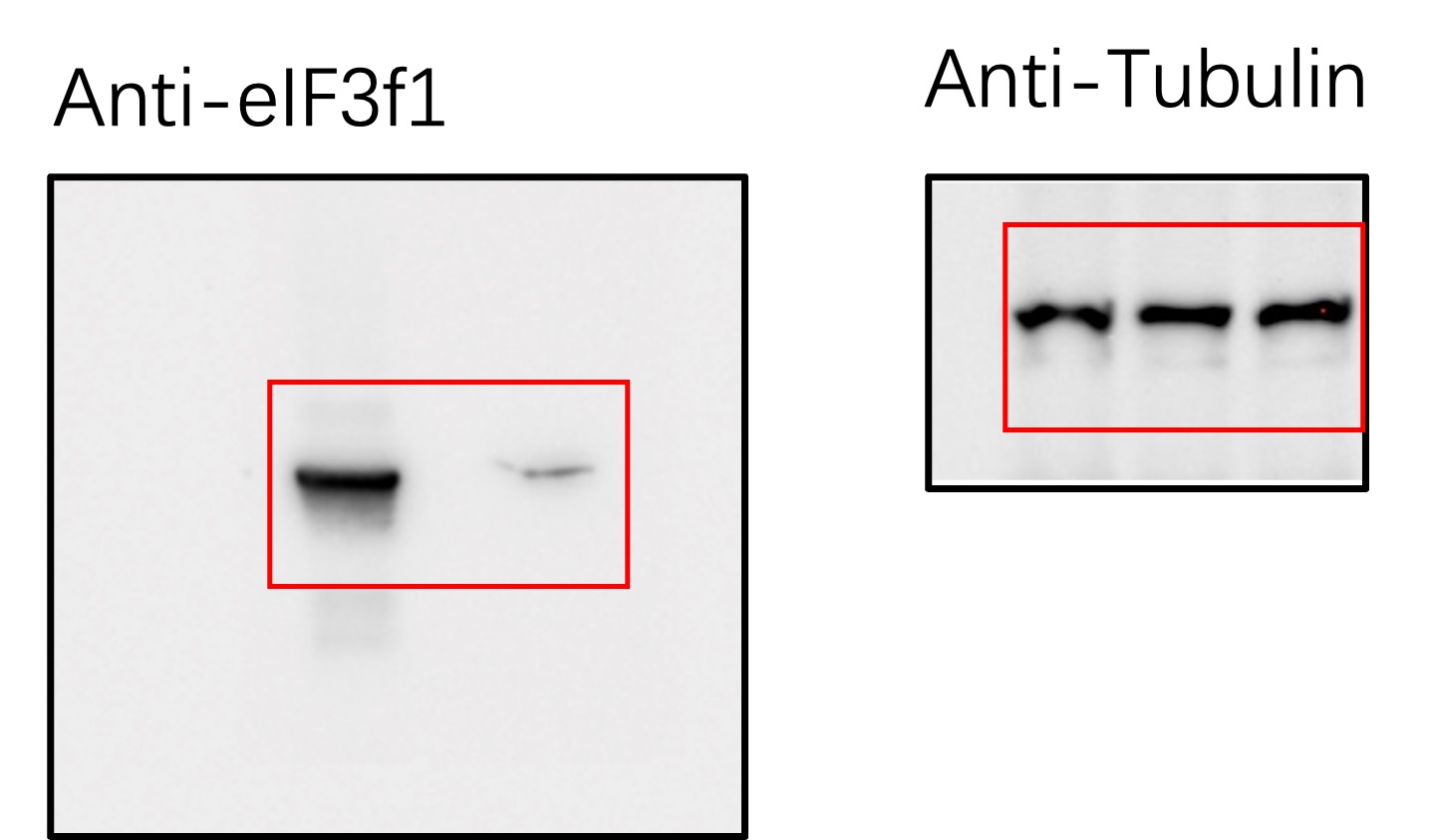

Supplement: Supplementary file 1 — Source Data Fig. 1 [file 44319_2024_67_MOESM1_ESM.zip › Figure 1/1I/Western blot.jpg]

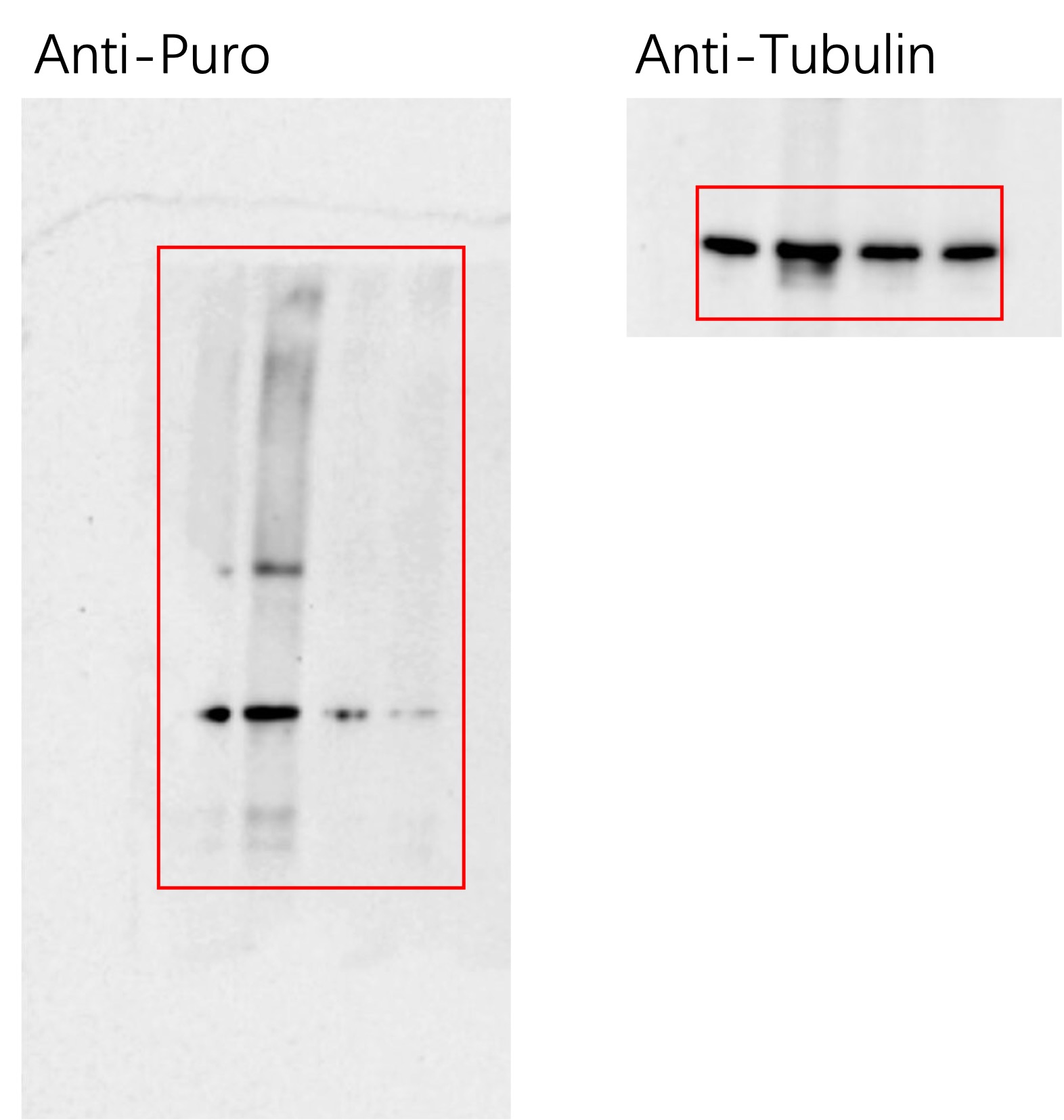

Supplement: Supplementary file 1 — Source Data Fig. 1 [file 44319_2024_67_MOESM1_ESM.zip › Figure 4/4A/Western blot.jpg]

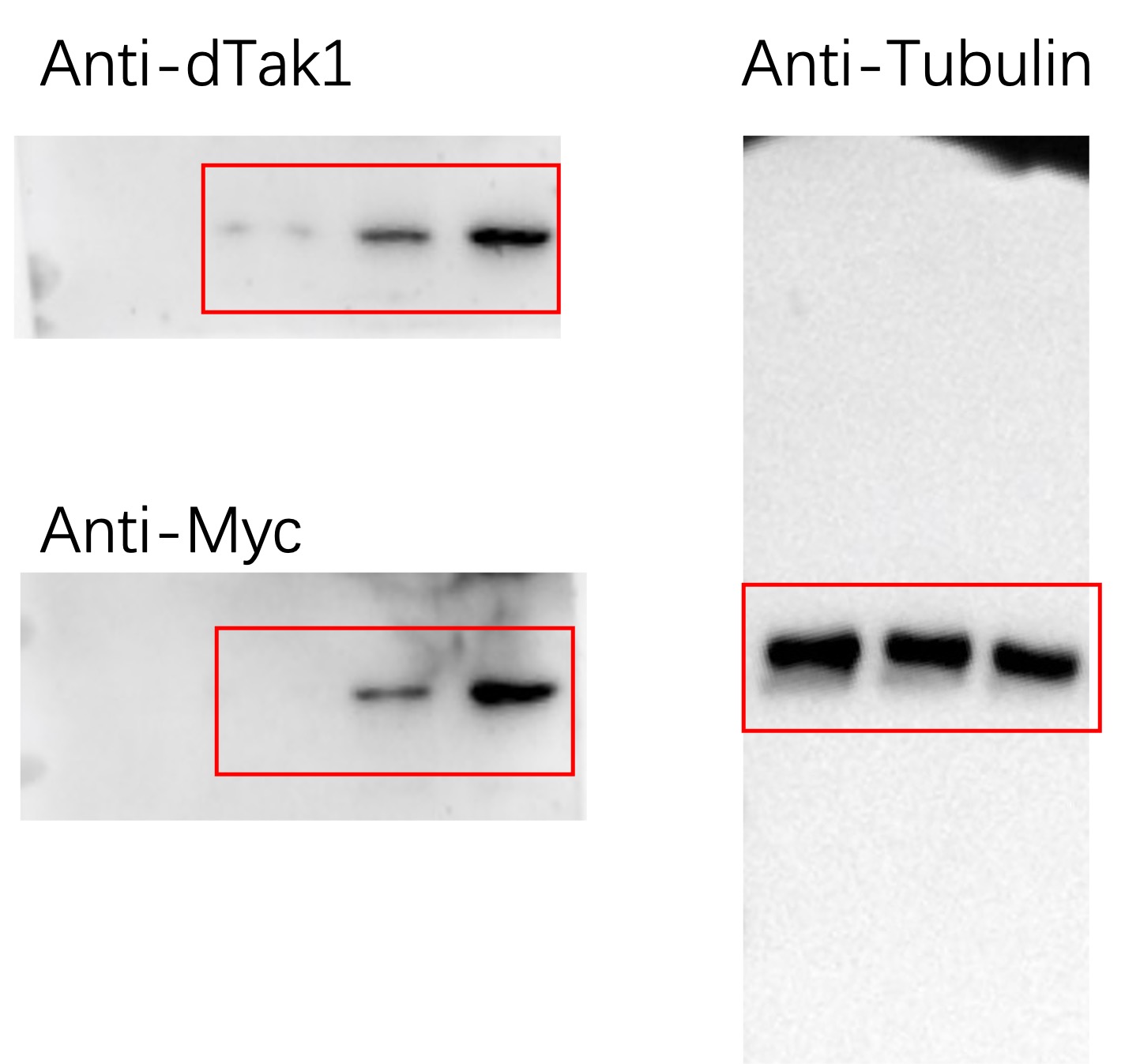

Supplement: Supplementary file 1 — Source Data Fig. 1 [file 44319_2024_67_MOESM1_ESM.zip › Figure 4/4E/Western blot.jpg]

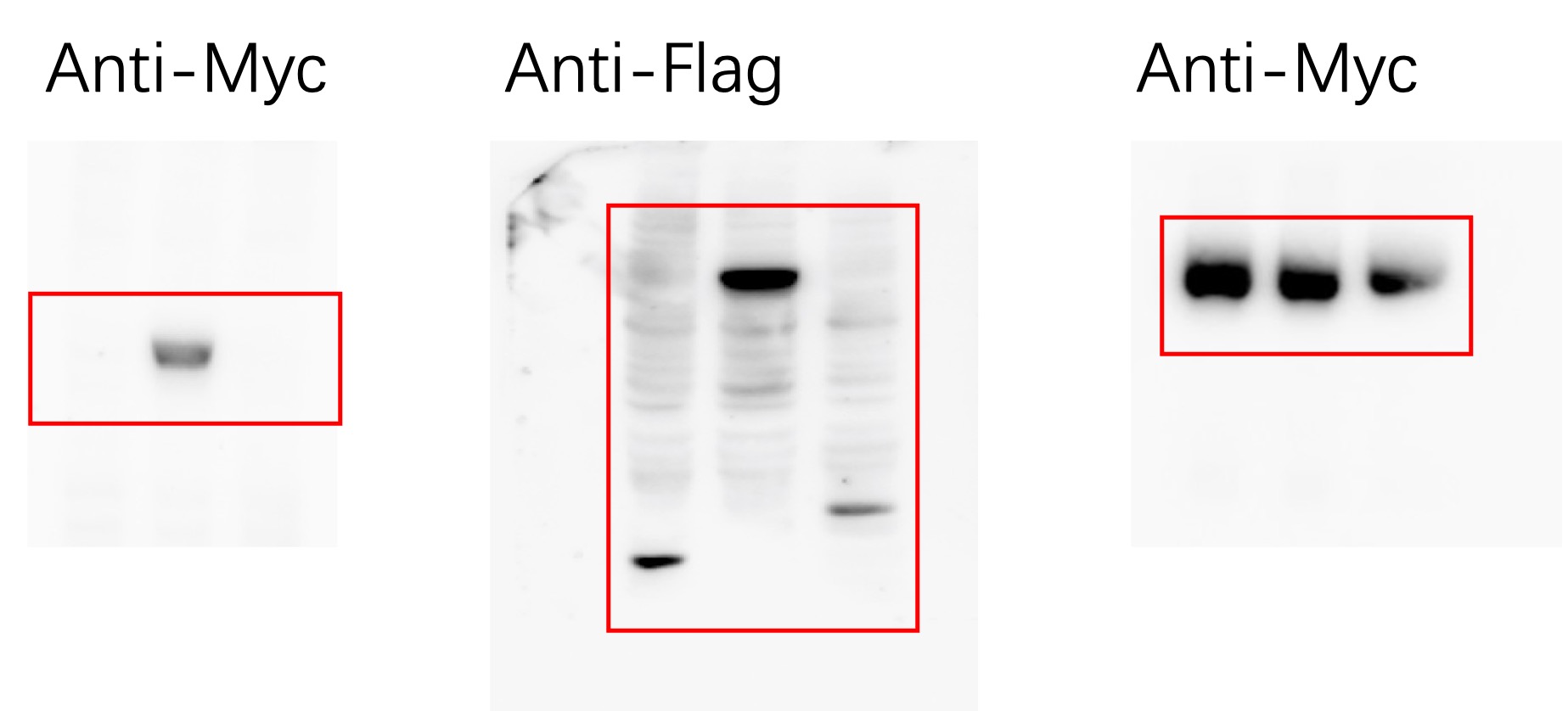

Supplement: Supplementary file 1 — Source Data Fig. 1 [file 44319_2024_67_MOESM1_ESM.zip › Figure 5/5A/Western blot.jpg]

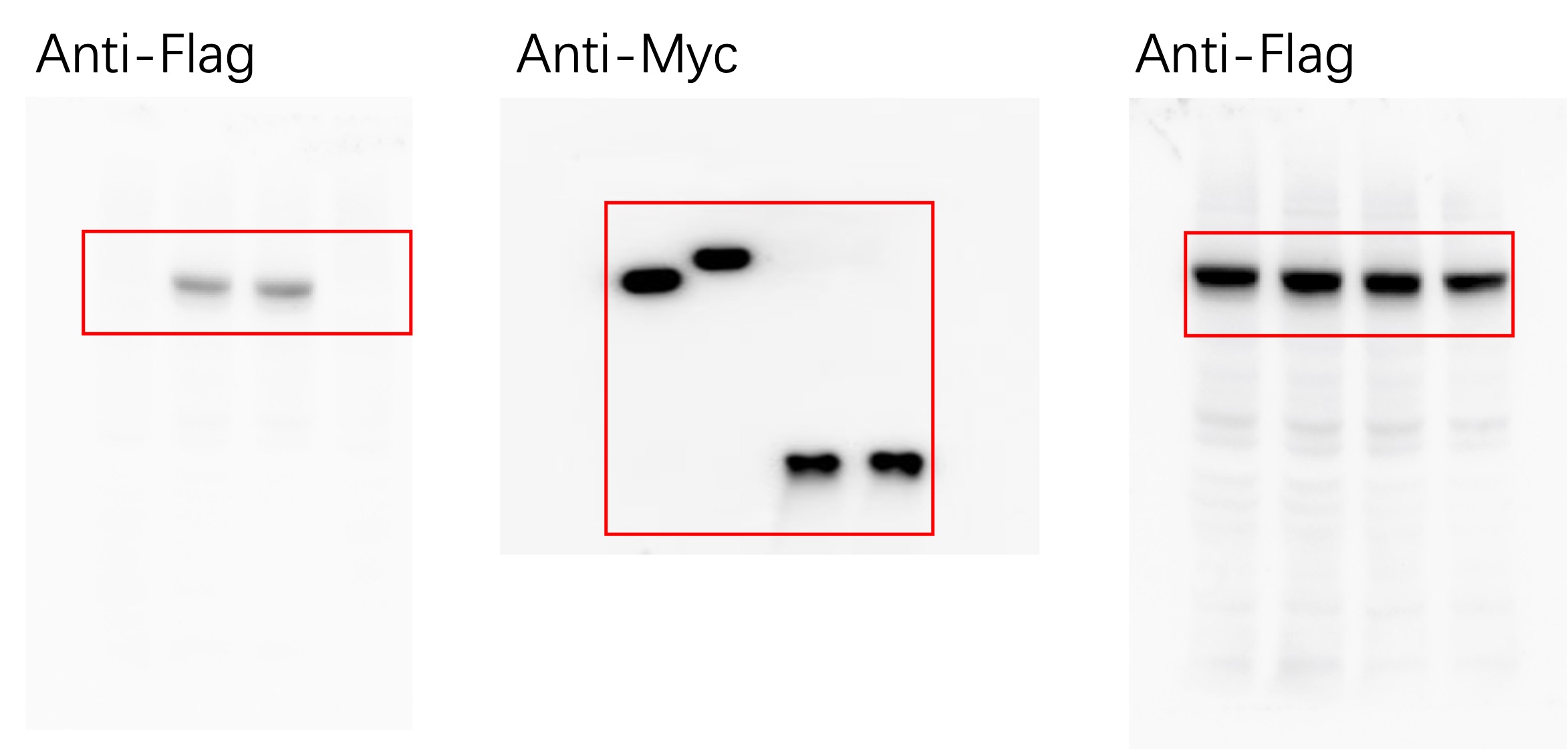

Supplement: Supplementary file 1 — Source Data Fig. 1 [file 44319_2024_67_MOESM1_ESM.zip › Figure 5/5B/Western blot.jpg]

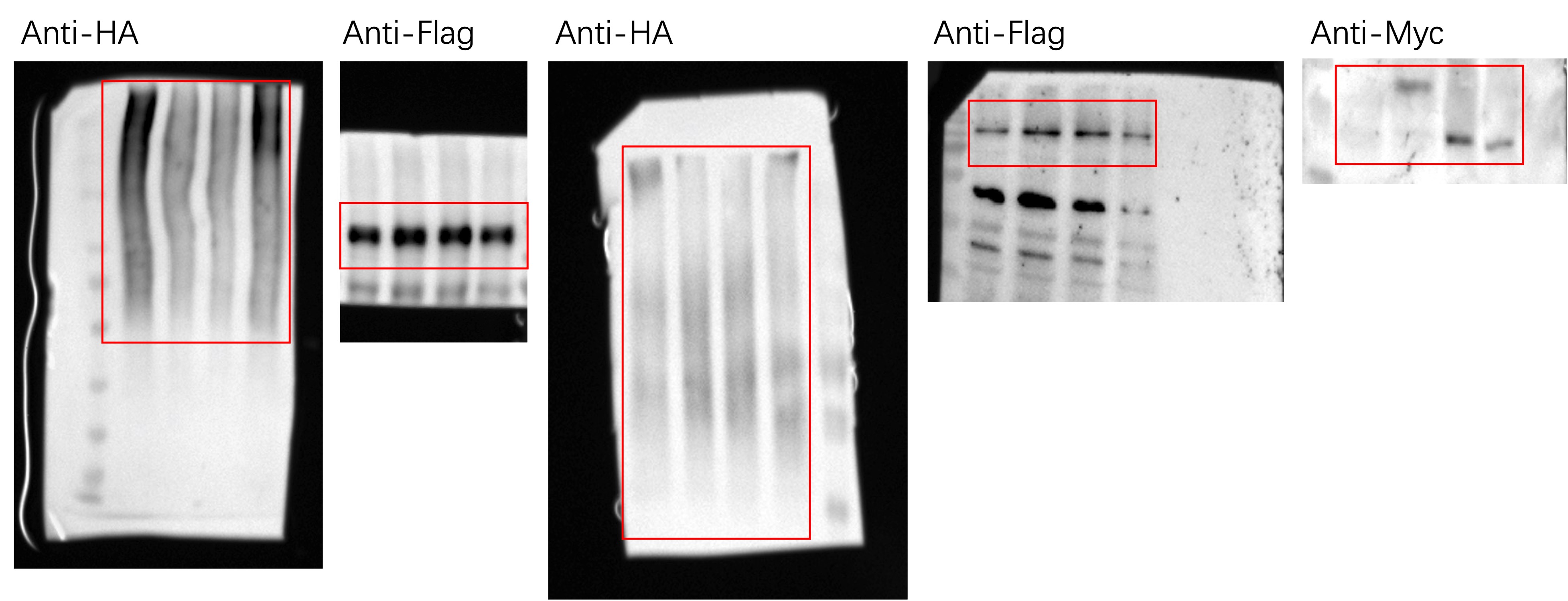

Supplement: Supplementary file 1 — Source Data Fig. 1 [file 44319_2024_67_MOESM1_ESM.zip › Figure 5/5D/Western blot.jpg]

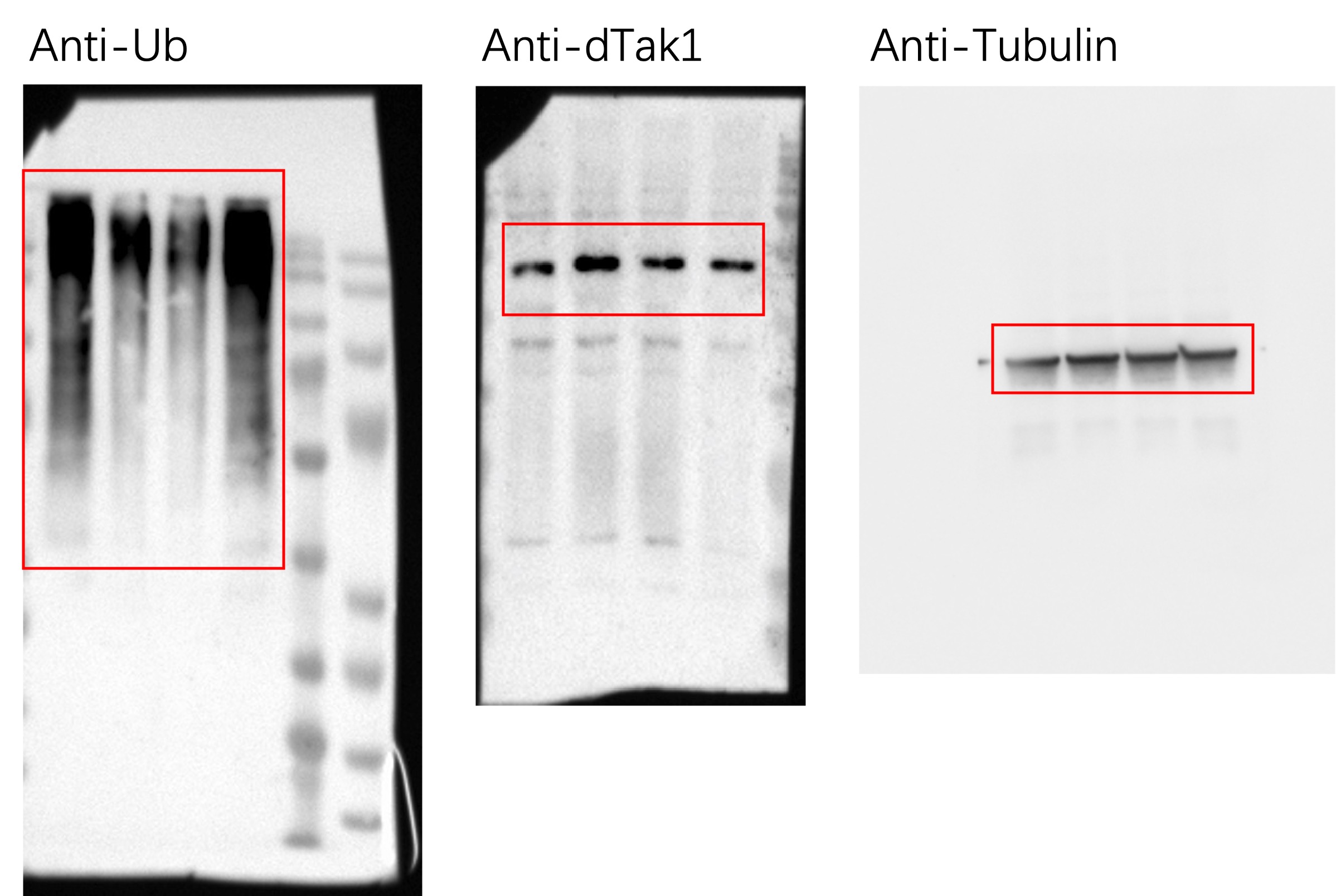

Supplement: Supplementary file 1 — Source Data Fig. 1 [file 44319_2024_67_MOESM1_ESM.zip › Figure 5/5F/Western blot.jpg]

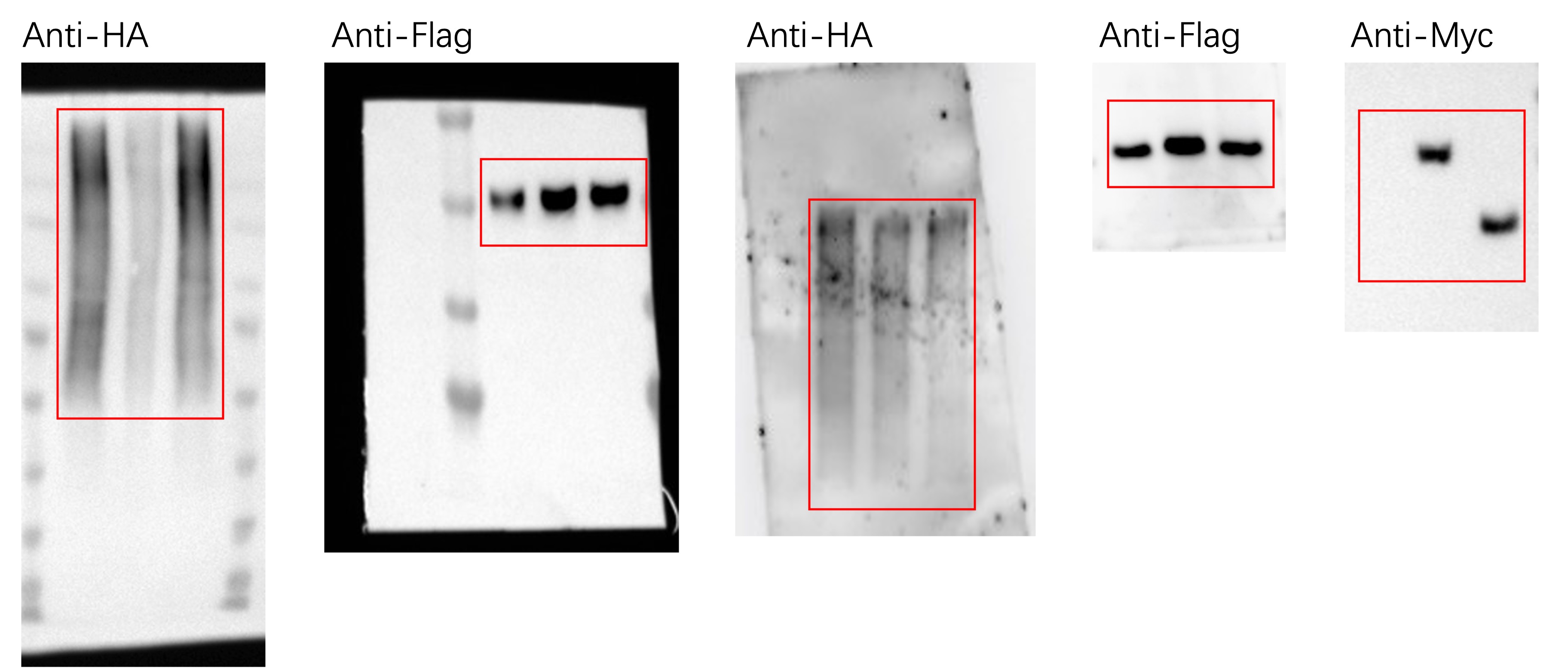

Supplement: Supplementary file 1 — Source Data Fig. 1 [file 44319_2024_67_MOESM1_ESM.zip › Figure 5/5H/Western blot.jpg]

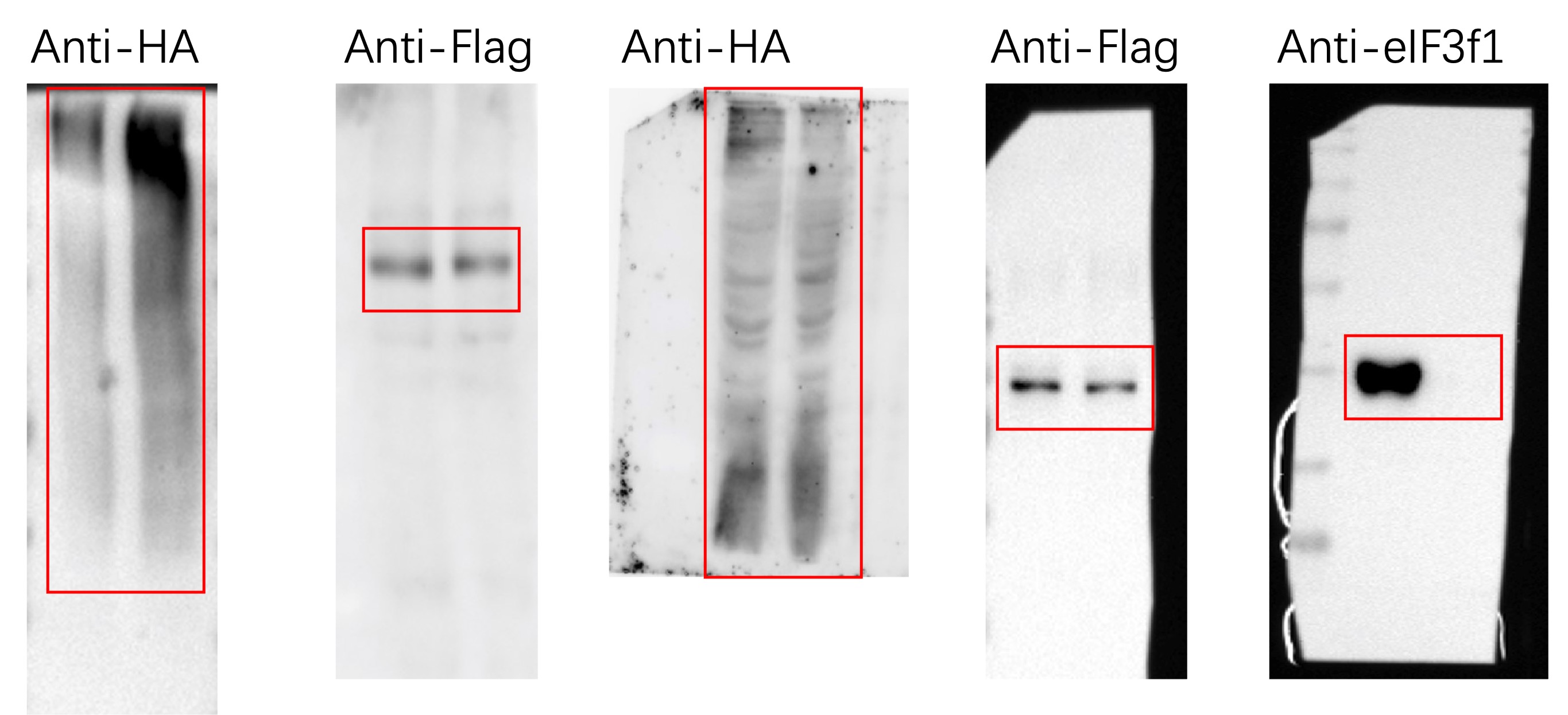

Supplement: Supplementary file 1 — Source Data Fig. 1 [file 44319_2024_67_MOESM1_ESM.zip › Figure 5/5J/Western blot.jpg]

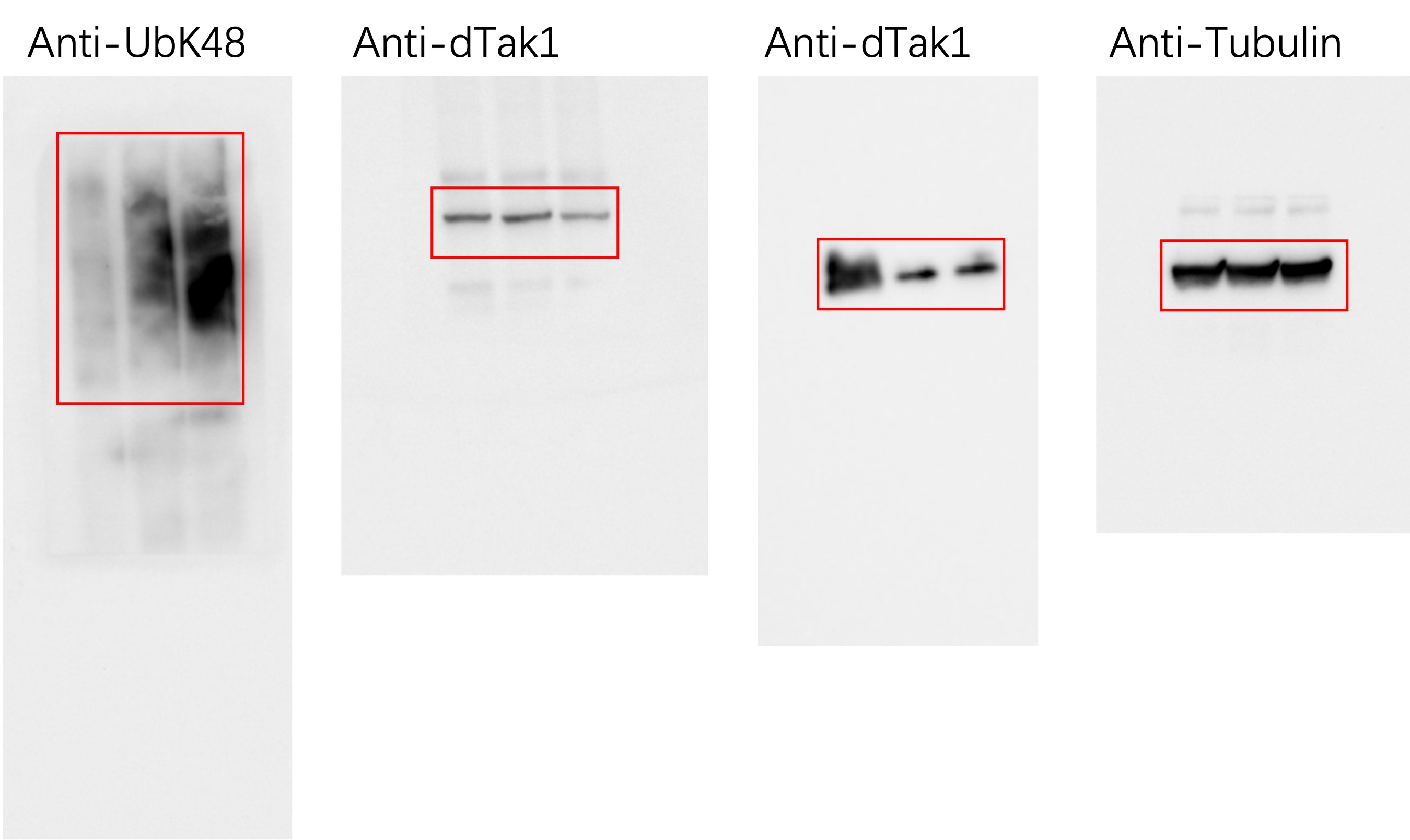

Supplement: Supplementary file 1 — Source Data Fig. 1 [file 44319_2024_67_MOESM1_ESM.zip › Figure 5/5L/Western blot.jpg]

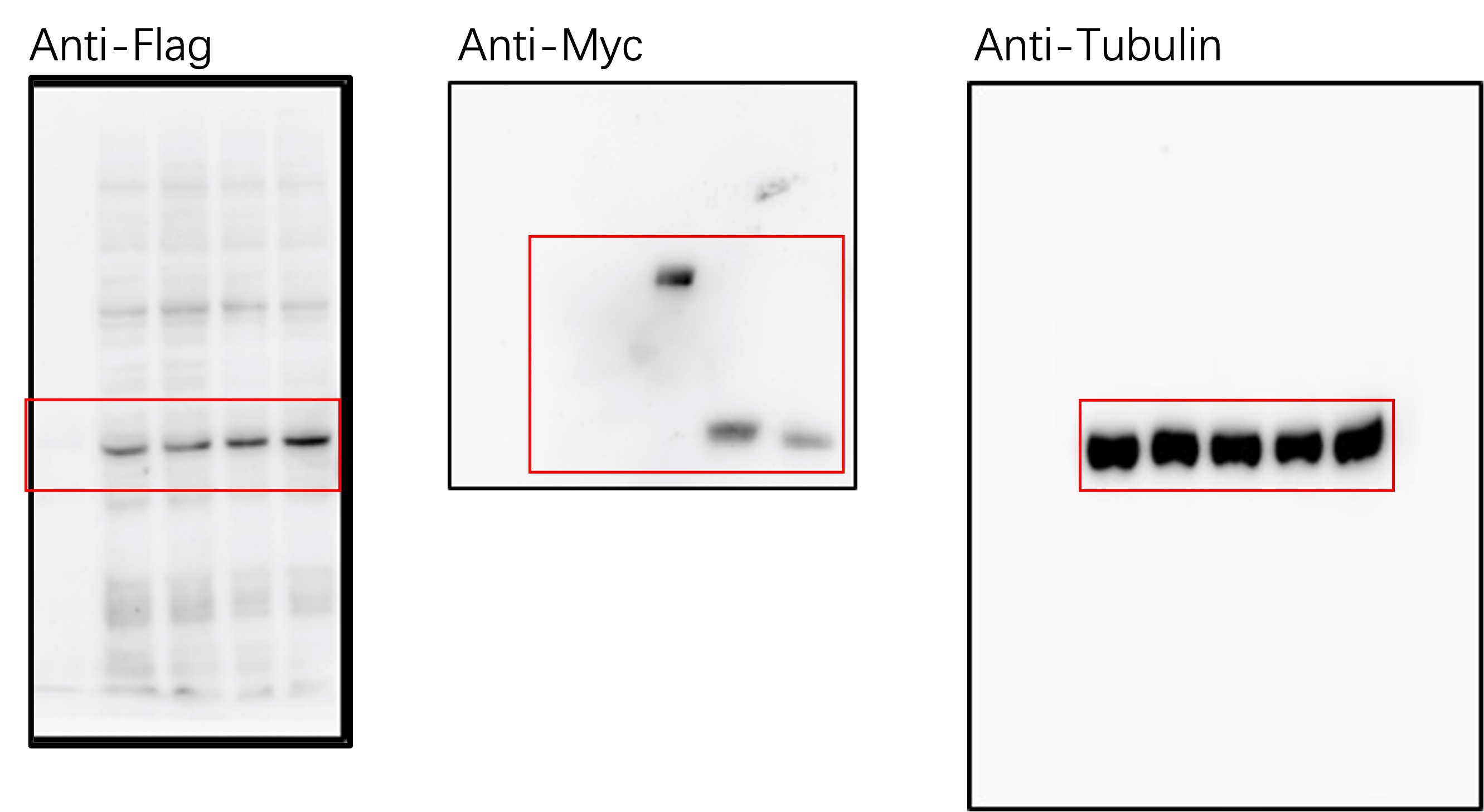

Supplement: Supplementary file 1 — Source Data Fig. 1 [file 44319_2024_67_MOESM1_ESM.zip › Figure 2/2B/Western blot.jpg]

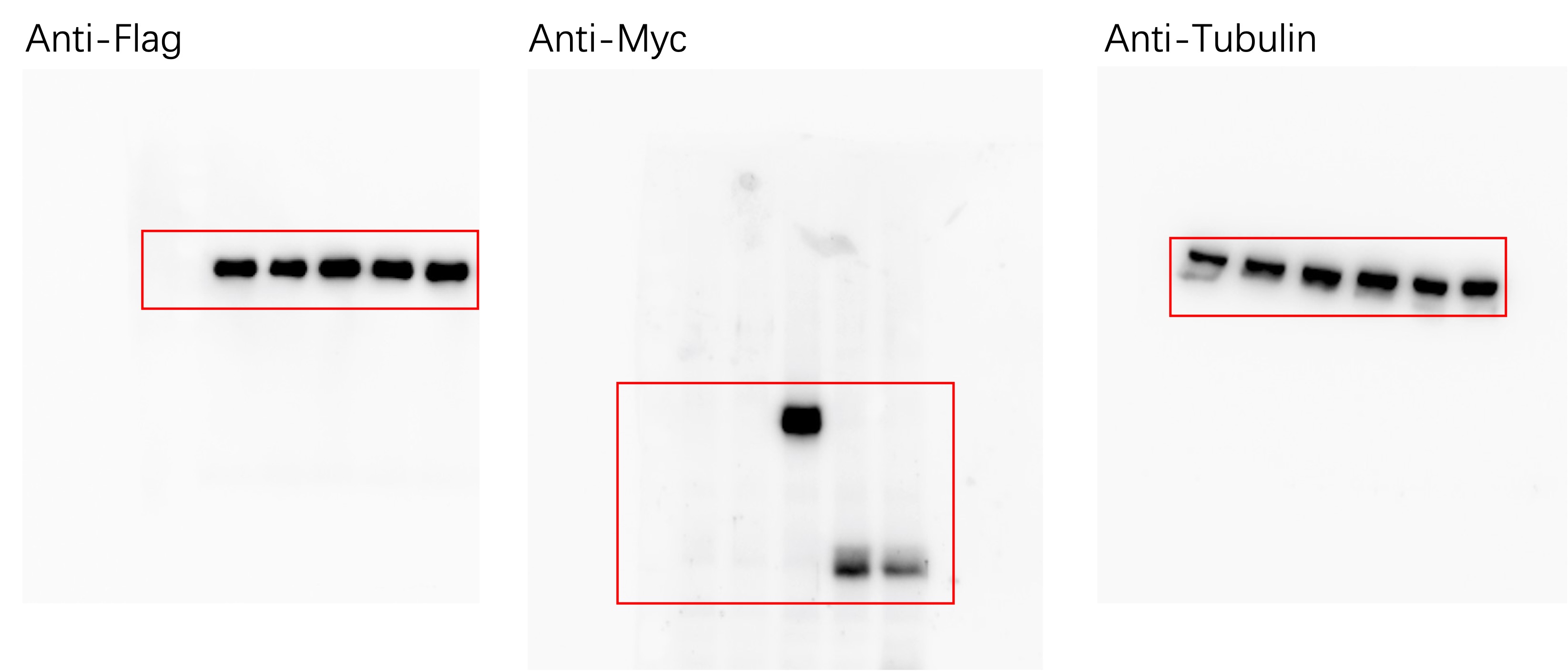

Supplement: Supplementary file 1 — Source Data Fig. 1 [file 44319_2024_67_MOESM1_ESM.zip › Figure 2/2F/Western blot.jpg]

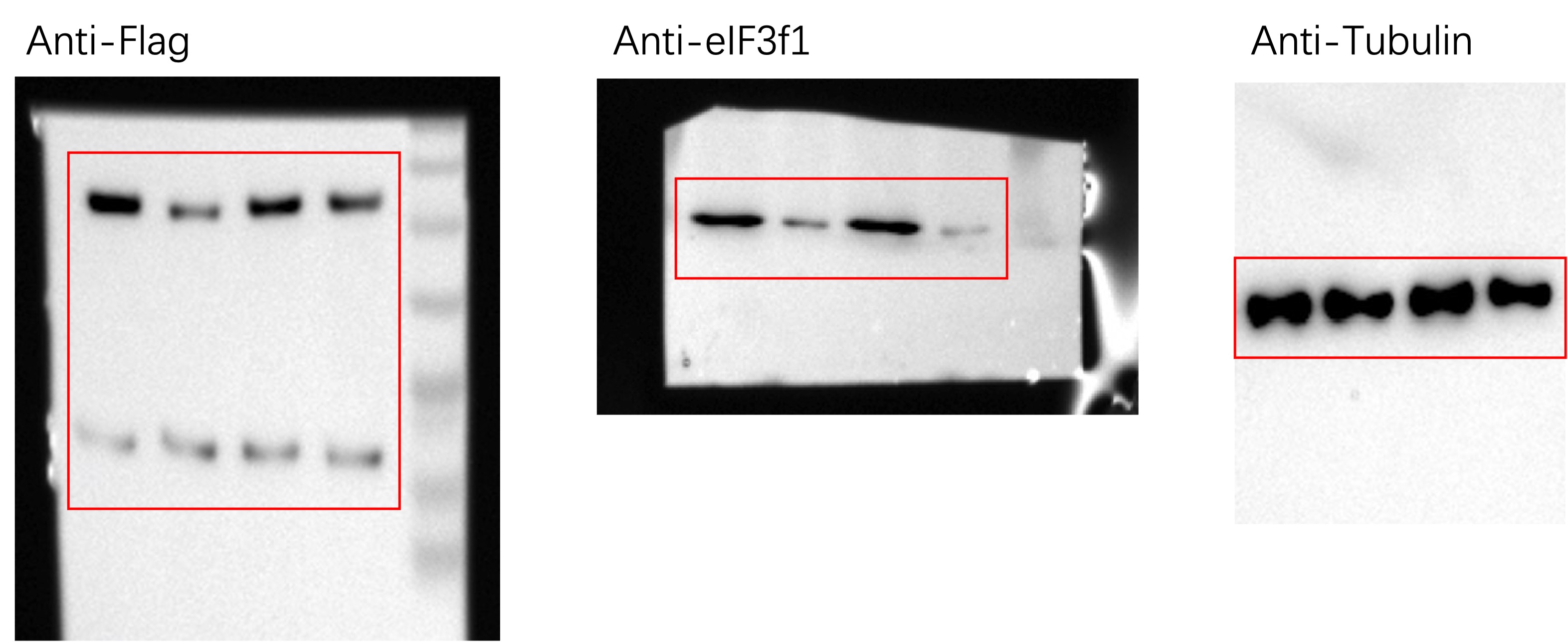

Supplement: Supplementary file 1 — Source Data Fig. 1 [file 44319_2024_67_MOESM1_ESM.zip › Figure 6/6A/Western blot.jpg]

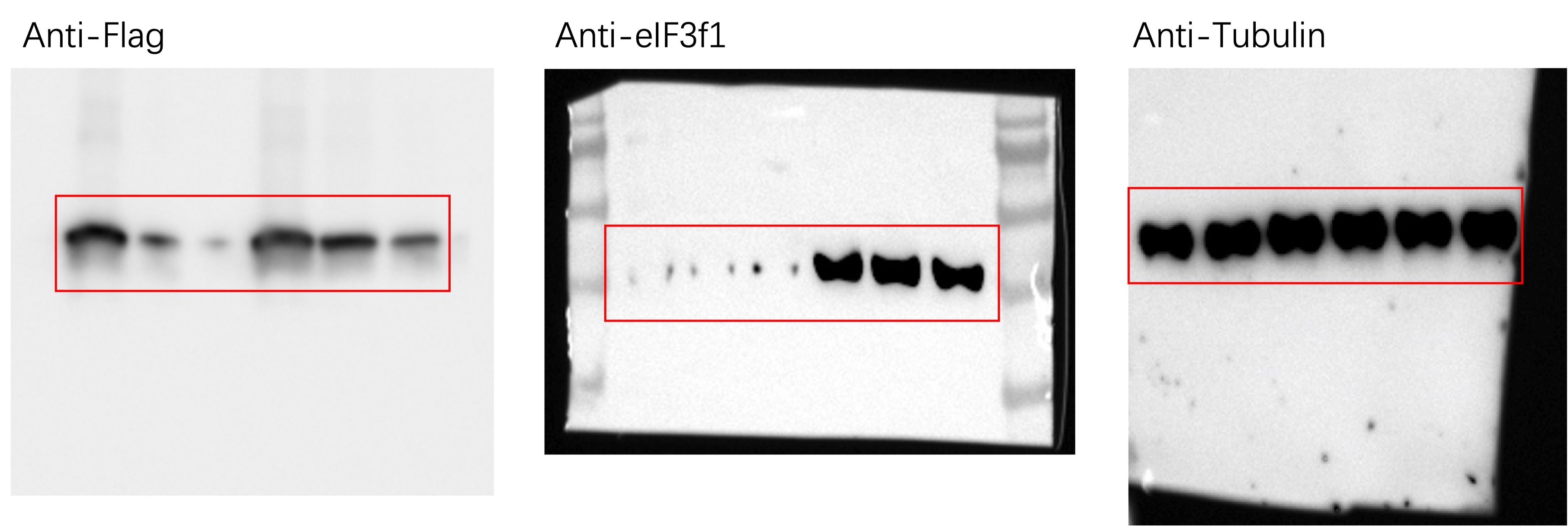

Supplement: Supplementary file 1 — Source Data Fig. 1 [file 44319_2024_67_MOESM1_ESM.zip › Figure 6/6C/Western blot.jpg]

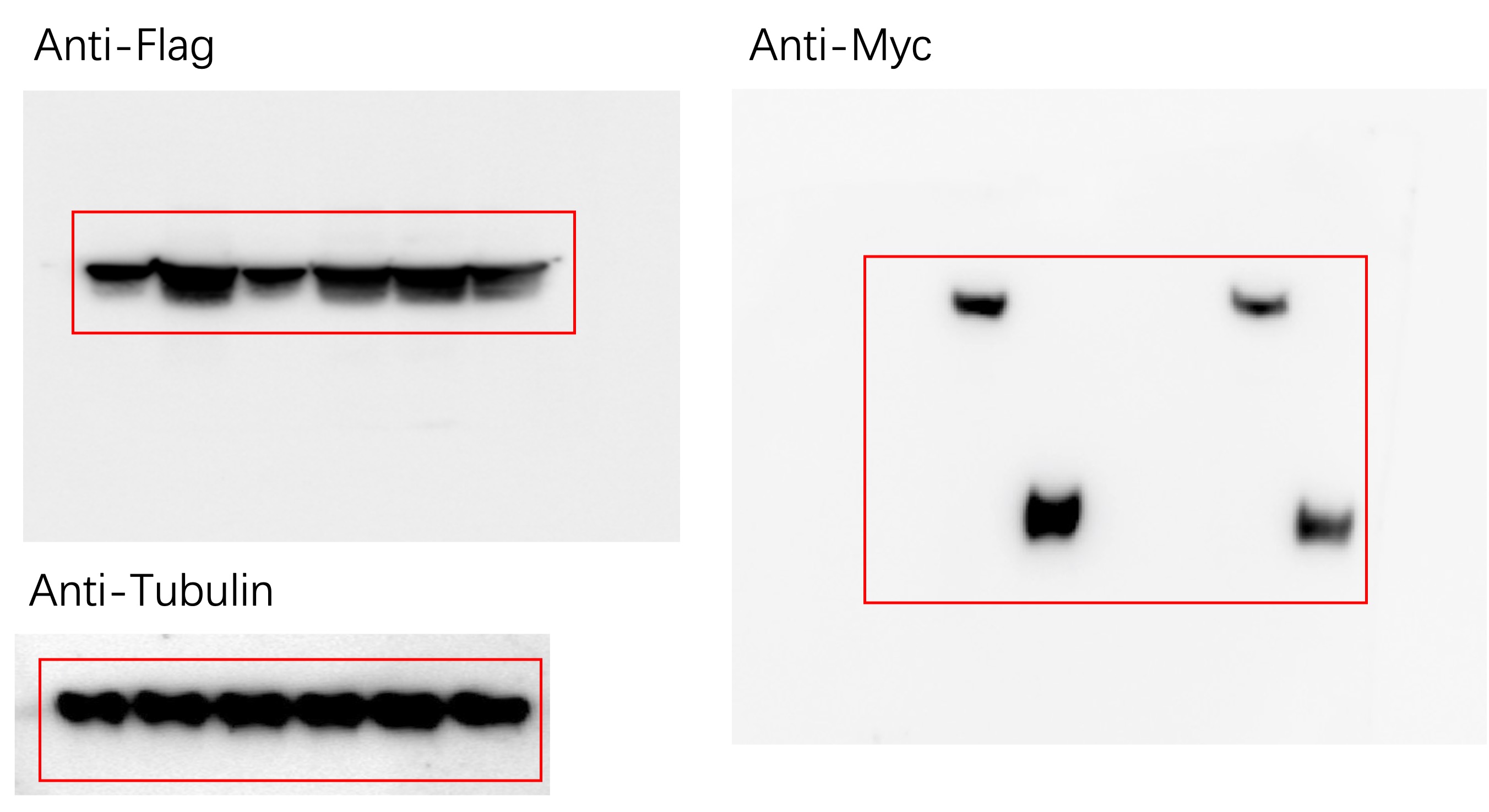

Supplement: Supplementary file 1 — Source Data Fig. 1 [file 44319_2024_67_MOESM1_ESM.zip › Figure 6/6E/Western blot.jpg]

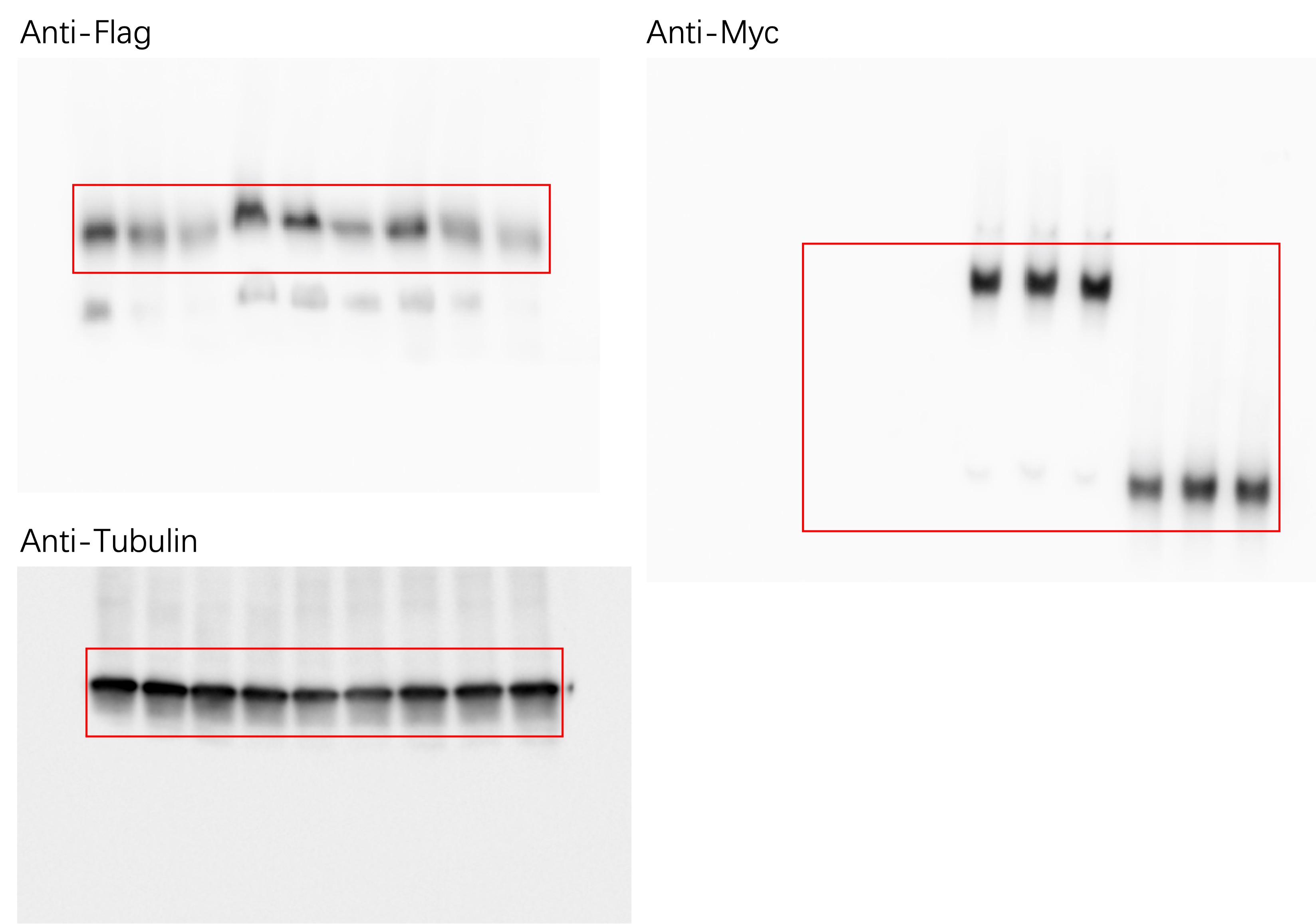

Supplement: Supplementary file 1 — Source Data Fig. 1 [file 44319_2024_67_MOESM1_ESM.zip › Figure 6/6G/Western blot.jpg]
