# Supplementary material for: Drosophila eIF3f1 mediates host immune defense by targeting dTak1
Source: EMBO Rep. 2024 Jan 26;25(3):26. doi: 10.1038/s44319-024-00067-z (PMC10933477; doi:10.1038/s44319-024-00067-z)
Supplement: Supplementary file 8 — Expanded View Figures [file 44319_2024_67_MOESM8_ESM.pdf]

Expanded View Figures

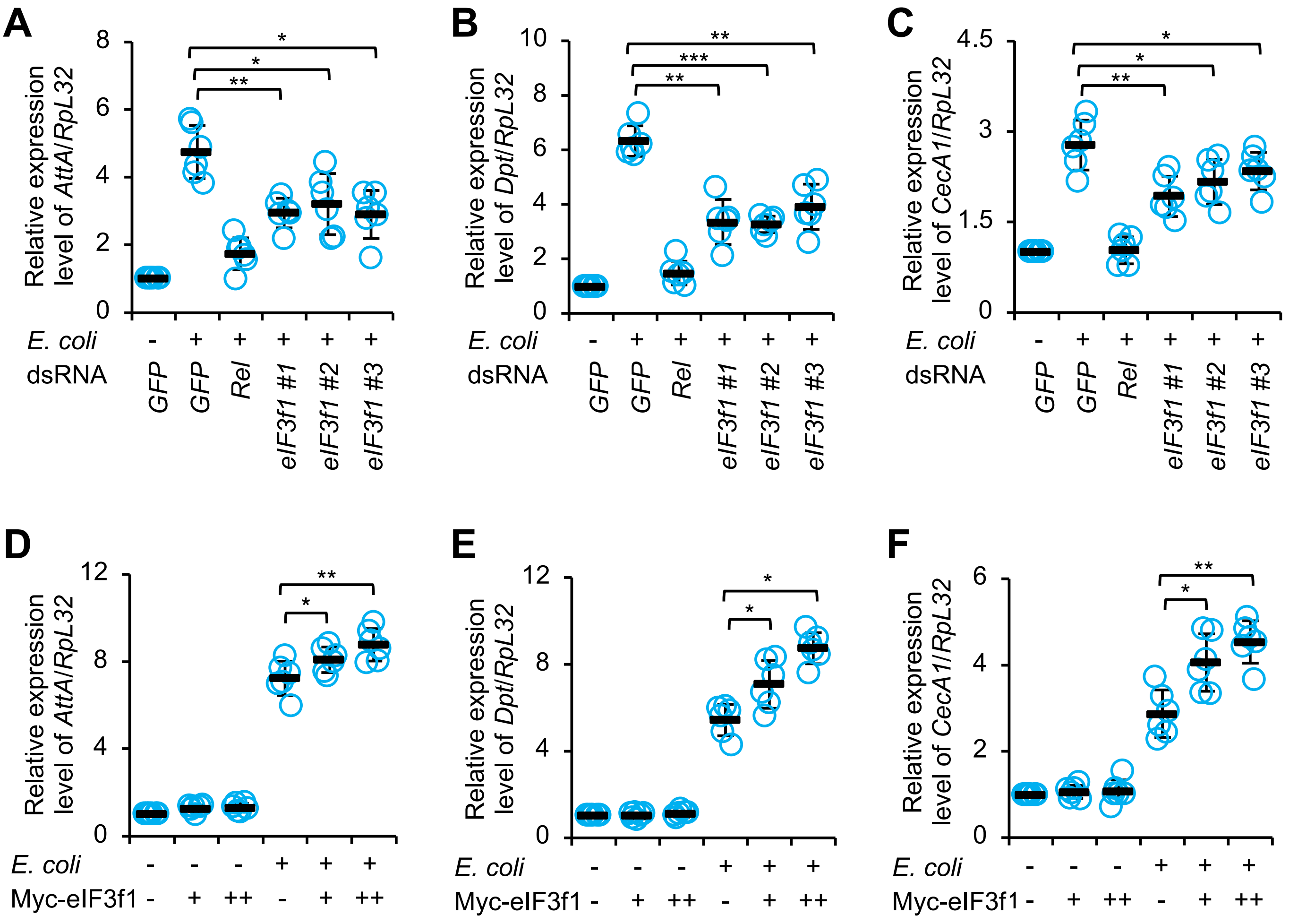

Figure EV1. ***eIF3f1* modulates IMD signaling in *Drosophila*.**

(**A–C**) S2 cells were treated with indicated dsRNAs for 48 h and then treated with heat-killed *E. coli* (MOI is around 5) for 6 h. Samples were subjected to RT-qPCR experiments to monitor the mRNA levels of *Att* (**A**), *Dpt* (**B**), or *CecA1* (**C**). (**D–F**) S2 cells were transfected with indicated expressing plasmids for 36 h, followed by treatment of heat-killed *E. coli* for 6 h. Samples were then subjected to RT-qPCR assays. Data Information: (**A–F**) Each dot represents one biological biological replicate and data are shown as mean ± SD. The nonparametric Kruskal–Wallis test was used for statistical analyses. **P* < 0.05; ***P* < 0.01; ****P* < 0.001.

© The Author(s)

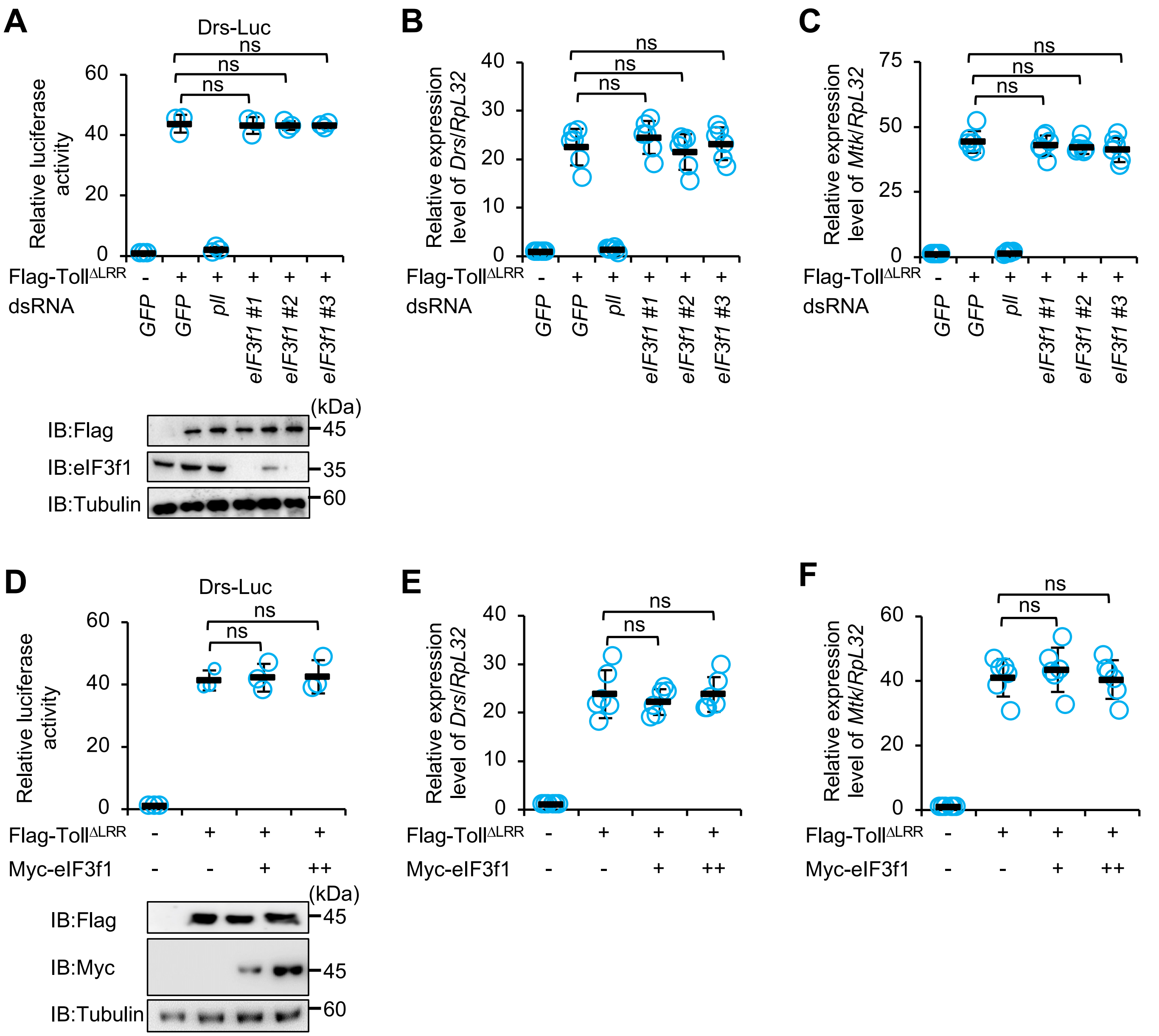

Figure EV2. *eIF3f1* does not affect Toll signaling in S2 cells.

(**A–C**) S2 cells were treated with indicated dsRNAs for 48 h. Cells were then transfected with various combinations of expressing plasmids for 36 h, followed by dual-luciferase assays (**A**) or RT-qPCR experiments to monitor the mRNA levels of *Drs* (**B**) or *Mtk* (**C**). (**D–F**) S2 cells were transfected with indicated expressing plasmids. 36 h post transfection, cells were harvested for dual-luciferase (**D**) or RT-qPCR (**E**, **F**) assays. Data Information: (**A–F**) Each dot represents one biological replicate and data are shown as mean ± SD. The nonparametric Kruskal–Wallis test was used for statistical analyses. ns, not significant, $P > 0.05$.

 © The Author(s)

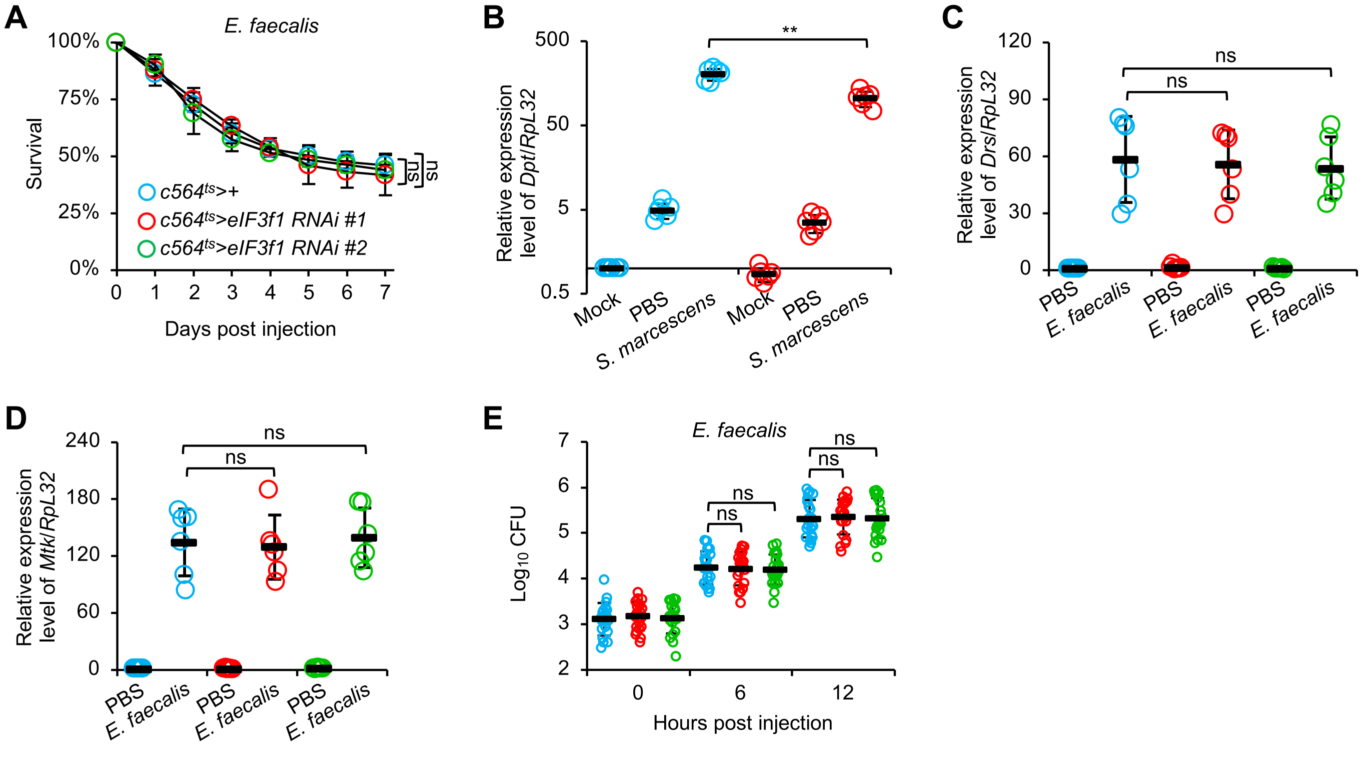

Figure EV3. *eIF3f1* is dispensable for the fly immune defense upon *E. faecalis* infection.

(**A–E**) Adult flies including *c564*^{ts} > +, *c564*^{ts} > *eIF3f1 RNAi #1*, and *c564*^{ts} > *eIF3f1 RNAi #2* were infected with *E. faecalis* (**A**, **C–E**), *S. marcescens* (**B**), PBS (**B–D**), or without treatment (mock in **B**). Flies were subjected to survival analysis (**A**), or RT-qPCR assays (6 h post-infection, **B–D**), or bacterial burden assay (**E**). Data Information: (**A**) Data are shown as mean ± SD and the Log-Rank test was used for statistical analyses. The numbers of flies are as follows. *c564*^{ts} > +: 89, 85, 89; *c564*^{ts} > *eIF3f1 RNAi #1*: 84, 87, 85; *c564*^{ts} > *eIF3f1 RNAi #2*: 90, 92, 84. (**B–E**) Each dot represents one biological replicate and data are shown as mean ± SD. The nonparametric Kruskal–Wallis test was used for statistical analyses. ***P* < 0.01; ns, not significant, *P* > 0.05.

© The Author(s)

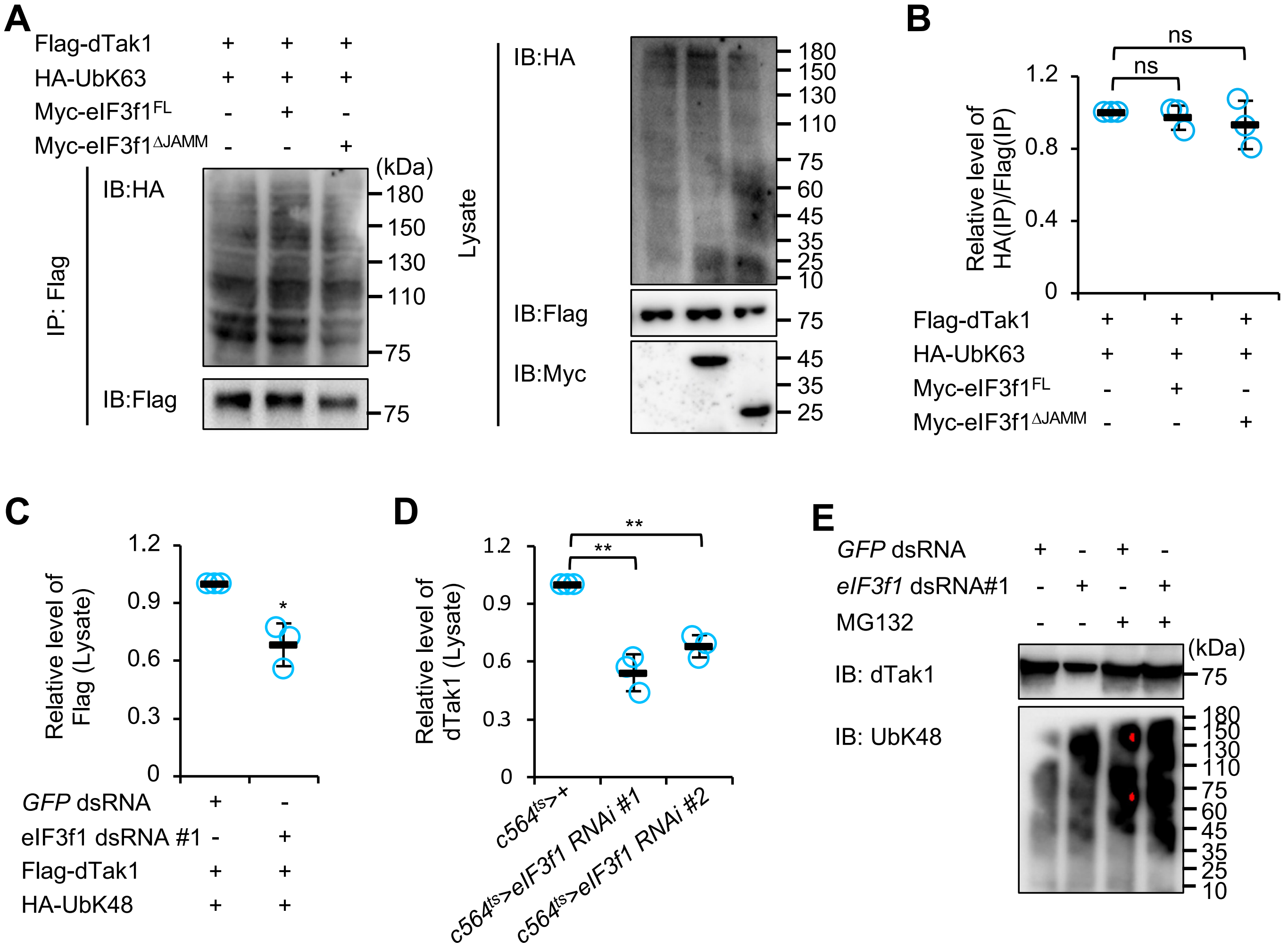

Figure EV4. eIF3f1 is not involved in regulating the K63-linked ubiquitination of dTak1.

(**A**, **B**) S2 cells were transfected with indicated combinations of expressing plasmids. 48 h post transfection, cells were harvested for ubiquitination assay (**A**). Densitometry analysis to quantify the K63-linked ubiquitination levels of dTak1 is shown in (**B**). (**C**) Densitometry analysis to quantify Flag-dTak1 expression level in Fig. 5J. (**D**) Densitometry analysis to quantify dTak1 expression level in Fig. 5L. (**E**) S2 cells were treated with *GFP* or *eIF3f1* dsRNAs for 48 h, followed by MG132 treatment for 6 h as indicated. Cells were lysed for western blot assays using anti-dTak1 and anti-UbK48 antibodies. Data Information: (**B**, **D**) Each dot represents one biological replicate and data are shown as mean ± SD. The nonparametric Kruskal–Wallis test was used for statistical analyses. (**C**) The Tukey's test was used for statistical analysis. $^{*}P < 0.05$; $^{**}P < 0.01$; ns, not significant, $P > 0.05$.

 © The Author(s)

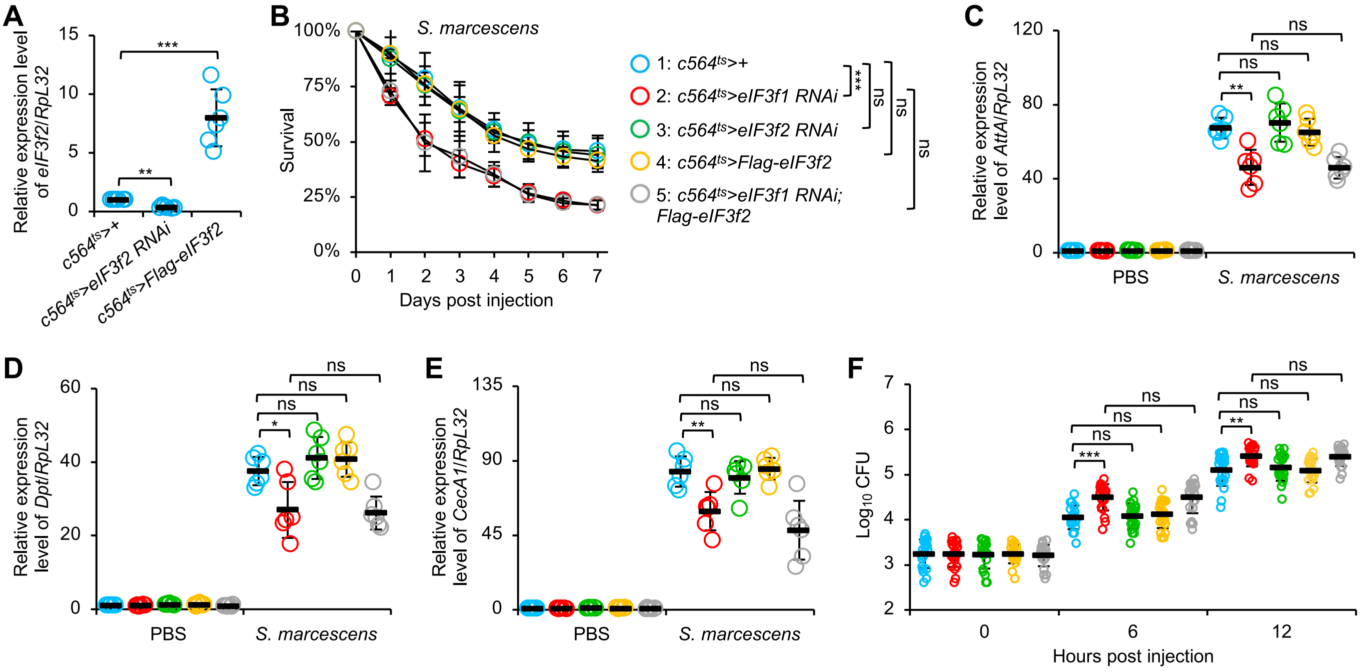

Figure EV5. ***eIF3f2* is not involved in mediating the fly immune defense upon microbial infection.**

(**A**) RT-qPCR assays examining the knockdown or overexpression effect of *eIF3f2*. (**B–F**) Adult flies including *c564*^{ts} > +, *c564*^{ts} > *eIF3f1 RNAi*, *c564*^{ts} > *eIF3f2 RNAi*, *c564*^{ts} > *Flag-eIF3f2*, and *c564*^{ts} > *eIF3f1 RNAi;Flag-eIF3f2* were infected with *S. marcescens* or PBS (control). Flies were subjected to survival analysis (**B**), or RT-qPCR assays (6 h post-infection, **C–E**), or bacterial burden assay (**F**). Data Information: (**A**, **C–F**) Each dot represents one biological replicate. Data are shown as mean ± SD. The nonparametric Kruskal–Wallis test was used for statistical analyses. (**B**) Data are shown as mean ± SD and the Log-Rank test was used for statistical analyses. The numbers of flies are as follows. *c564*^{ts} > +: 89, 88, 94; *c564*^{ts} > *eIF3f1 RNAi*: 91, 89, 84; *c564*^{ts} > *eIF3f2 RNAi*: 96, 87, 89; *c564*^{ts} > *Flag-eIF3f2*: 88, 89, 89; *c564*^{ts} > *eIF3f1 RNAi;Flag-eIF3f2*: 89, 93, 94. *P < 0.05; **P < 0.01; ***P < 0.001; ns, not significant, P > 0.05.

© The Author(s)
